# Editorial: Advancing plant abiotic stress research with integrated multi-omics technologies

**DOI:** 10.3389/fpls.2026.1825165

**Published:** 2026-04-01

**Authors:** Prateek Gupta, Dilip Kumar, Kamal Tyagi, Deepanker Yadav

**Affiliations:** 1 Department of Biological Sciences, SRM University-AP, Andhra Pradesh, Guntur, India; 2 Laboratory of Post-Transcriptional Control of Gene Expression, Institute of Microbiology of the Czech Academy of Sciences, Prague, Czechia; 3 Horticulture Section, College of Agriculture and Life Sciences, Cornell University, Ithaca, NY, United States; 4 Department of Botany, Guru Ghasidas Vishwavidyalaya (A Central University), Bilaspur, Chhattisgarh, India

**Keywords:** abiotic stress, crop improvement, functional genomics, metabolic reprograming, metabolomics, signalling, stress response, transcriptomics

## Introduction

Abiotic stresses like drought, salinity, extreme temperatures, waterlogging, and heavy-metal toxicity have always been major limiting factors in crop productivity worldwide. These stresses affect the normal physiological and metabolic activities and lead to reduced crop productivity and quality. Current data suggests that abiotic stress has been found to cause losses in crop yield by around 50% globally, posing a major threat to sustainable agriculture (Nehra et al., 2024).

Plants have evolved sophisticated mechanisms to cope with adverse environmental conditions through adaptation at the molecular, cellular, and whole-plant levels. These responses involve transcriptional regulation, hormonal signalling pathways, metabolic reprogramming, and antioxidant defence systems (Zhao et al., 2025; Khan, 2025). Despite considerable advances in the field of plant stress biology, it has been observed that complex adaptation mechanisms in plants are regulated through interconnected networks of genes, proteins and metabolites. Therefore, classical molecular studies focusing on individual genes and pathways are often insufficient to fully understand these complex adaptive mechanisms.

Recent advances have led to the application of multi-omics technologies involving genomics, transcriptomics, metabolomics, proteomics and microbiomics in relation to crop research. Such integrated approaches are useful in studying complex adaptation mechanisms in relation to environmental stresses in plants (Saleem et al., 2025; Roychowdhury et al., 2023).

The Research Topic, “Advancing Plant Abiotic Stress Research with Integrated Multi-Omics Technologies,” was conceived to emphasize the latest achievements in the application of multi-omics technologies for exploring and understanding the response of plants to abiotic stresses. The nine research articles included in this special issue showcase the wide-ranging applications of integrative omics technologies in different plants and various abiotic stress conditions.

## Multi-omics insights into drought stress responses

Drought stress, one of the most limiting abiotic stresses, affects plants in almost all parts of the world. Plants respond to water deficit through a series of coordinated physiological, biochemical, and molecular adjustments that enable them to survive under limited water availability. These responses include physiological adaptations such as osmotic adjustment for maintaining cellular water balance, activation of antioxidant defence systems to mitigate oxidative damage, and large-scale transcriptional reprogramming that regulates stress-responsive genes (Fang and Xiong, 2015).

Several studies included in this Research Topic focus on elucidating the mechanisms of drought tolerance using integrated omics approaches. Han et al. employed combined transcriptomic and metabolomic analyses to investigate genotype-specific drought tolerance strategies in cotton. Their study revealed coordinated changes in gene expression and metabolite accumulation under drought stress, particularly in pathways associated with flavonoid and phenylpropanoid biosynthesis. The analysis further identified several hub regulatory genes that may play important roles in enhancing drought adaptation in cotton.

Similarly, Ye et al. investigated drought responses in the medicinal plant *Indigofera stachyodes* using integrated transcriptomic and metabolomic analyses. They identified various metabolic pathways related to drought adaptation in roots. The results emphasized the flavonoid and phenylpropanoid pathway in relation to drought stress. suggesting their possible role in maintaining cellular stability under water-deficient conditions.

As a complement to the above findings, Wang et al. examined drought resistance mechanisms in different sugarcane varieties through transcriptomics combined with co-expression network analysis. Their study identified several transcription factor families, including NAC, MYB, ERF and bZIP, that appear to regulate drought-responsive gene expression. In addition, several hub genes associated with photosynthesis, hormone signalling and antioxidant defence were also identified, indicating their involvement in drought tolerance mechanisms in sugarcane.

Aside from molecular-based investigations on drought stress in plants, one study included in this Research Topic focused on strategies for mitigating drought stress. Abdolmaleki et al. examined the effects of superabsorbent polymer seed coatings on drought tolerance in rapeseed. Using a combination of physiological measurements and transcriptomic analysis, the study showed that specific polymer formulations influenced the expression of several stress-related genes and improved seedling establishment under water-limited conditions. These findings suggest that integrating physiological approaches with omics analyses can provide useful insights for developing strategies to improve drought resilience in crops.

## Expanding the scope: salinity, waterlogging, and heavy-metal stress

While drought represents one of the most widely studied abiotic stresses, plants in natural environments are often exposed to multiple stress factors simultaneously. Therefore, understanding plant responses to other abiotic stresses is equally important. Integrated multi-omics technologies have increasingly been applied to investigate these complex stress responses.

Salinity stress is another major constraint affecting large areas of irrigated agricultural land worldwide (Munns and Tester, 2008). In this Research Topic, Ren et al. investigated salt-tolerant germination in highland barley by combining transcriptome sequencing with 16S rRNA-based microbial community analysis. Their results suggested that several processes, including ion homeostasis, regulation of oxidative stress and interactions with endophytic bacteria, contribute to improved salt tolerance during germination.

Waterlogging is another important abiotic stress that reduces oxygen availability in the root zone and affects plant metabolism. Fang et al. analysed waterlogging responses in *Magnolia sinostellata* using integrated transcriptomic and metabolomic approaches. Their study indicated extensive transcriptional and metabolic changes across plant tissues, with phytohormone signalling pathways playing important roles in regulating adaptive morphological responses such as adventitious root formation and aerenchyma development.

Heavy-metal contamination also represents a growing environmental challenge affecting agricultural ecosystems. Yang et al. studied the influence of silica nanoparticles on cadmium toxicity in pea seedlings. The results suggested that nanoparticle treatment reduced oxidative stress and influenced carbon and nitrogen metabolism, which may contribute to improved tolerance to cadmium exposure.

## Regulatory networks and emerging signalling mechanisms

In addition to understanding the mechanisms that govern stress tolerance in plants, there is a need to gain further insight into the genetic and molecular regulators that are involved in stress adaptation mechanisms in plants. Liu et al., addressed this issue by conducting a comprehensive analysis on the pan-genome of the NAC transcription factors in barley plants. The study provided new insights into the role that transcription factors play in stress adaptation mechanisms in plants.

In addition to the above studies, Lachica et al., conducted a comprehensive review on the role that β-cyclocitral, a carotenoid-derived signalling molecule, plays in abiotic stress response mechanisms in plants. The review provided further insights into the emerging role that apocarotenoids play in stress response mechanisms in plants, where the molecules are known to play a role in regulating oxidative stress response, root development, and detoxification mechanisms in plants.

## Future perspectives for multi-omics-driven crop improvement

The studies included in the present Research Topic show the impact of the application of multi-omics approaches in the field of plant stress biology. Integrating multiple omics approaches enables a deeper understanding of the mechanisms underlying plant responses to abiotic stresses.

In the future, the application of different ‘omics’ approaches in combination with powerful computational tools, systems biology approaches, and gene editing technologies will likely facilitate the identification of the key players involved in the response of plants to different types of environmental stresses. Integrating multi-omics approaches with modern crop improvement strategies could facilitate the development of crops capable of performing well under diverse environmental conditions.
